# The impact of multimorbidity on Quality of Life in inflammatory myopathies: A cluster analysis from the COVAD dataset

**DOI:** 10.1093/rheumatology/keae520

**Published:** 2024-09-25

**Authors:** Marco Fornaro, Vincenzo Venerito, Maria Rosa Pellico, Florenzo Iannone, Mrudula Joshi, Yi-Ming Chen, Ai Lyn Tan, Sreoshy Saha, Tulika Chatterjee, Vishwesh Agarwal, Samuel Katsuyuki Shinjo, Leonardo Santos Hoff, Esha Kadam, Nelly Ziade, Tsvetelina Velikova, A T M Tanveer Hasan, Russka Shumnalieva, Marcin Milchert, Chou Luan Tan, Abraham Edgar Gracia-Ramos, Lorenzo Cavagna, Binit Vaidya, Masataka Kuwana, Syahrul Sazliyana Shaharir, Johannes Knitza, Ashima Makol, Erick Adrian Zamora Tehozol, Jorge Rojas Serrano, Hussein Halabi, Dzifa Dey, Carlos Enrique Toro-Gutiérrez, Phonpen Akarawatcharangura Goo, Carlo V Caballero-Uribe, Oliver Distler, Wanruchada Katchamart, Jessica Day, Ioannis Parodis, Elena Nikiphorou, Hector Chinoy, Vikas Agarwal, Latika Gupta, CoVAD Study Group, Parikshit Sen, Mahnoor Javaid, Laura Andreoli, Daniele Lini, Karen Schreiber, Arvind Nune, Ai Lyn Tan, Aarat Patel, John D Pauling, Chris Wincup, Bhupen Barman, Ignacio García-De La Torre, Iris J Colunga-Pedraza, Javier Merayo-Chalico, Okwara Celestine Chibuzo, Lina El Kibbi, James B Lilleker, Babur Salim, Tamer Gheita, Miguel A Saavedra, Zoltán Griger, Sinan Kardes, Melinda Nagy Vince, Yogesh Preet Singh, Rajiv Ranjan, Avinash Jain, Sapan C Pandya, Rakesh Kumar Pilania, Aman Sharma, Manesh Manoj M, Vikas Gupta, Chengappa G Kavadichanda, Pradeepta Sekhar Patro, Sajal Ajmani, Sanat Phatak, Rudra Prosad Goswami, Abhra Chandra Chowdhury, Ashish Jacob Mathew, Padnamabha Shenoy, Ajay Asranna, Keerthi Talari Bommakanti, Anuj Shukla, Arunkumar R Pande, Kunal Chandwar, Akanksha Ghodke, Hiya Boro, Zoha Zahid Fazal, Binit Vaidya, Döndü Üsküdar Cansu, Reşit Yıldırım, Armen Yuri Gasparyan, Nicoletta Del Papa, Gianluca Sambataro, Atzeni Fabiola, Marcello Govoni, Simone Parisi, Elena Bartoloni Bocci, Gian Domenico Sebastiani, Enrico Fusaro, Marco Sebastiani, Luca Quartuccio, Franco Franceschini, Pier Paolo Sainaghi, Giovanni Orsolini, Rossella De Angelis, Maria Giovanna Danieli, Silvia Grignaschi, Alessandro Giollo, Alessia Alunno, Lisa S Traboco, Suryo Anggoro Kusumo Wibowo, Jesús Loarce-Martos, Sergio Prieto-González, Raquel Aranega Gonzalez, Akira Yoshida, Ran Nakashima, Shinji Sato, Naoki Kimura, Yuko Kaneko, Takahisa Gono, Stylianos Tomaras, Fabian Nikolai Proft, Marie-Therese Holzer, Margarita Aleksandrovna Gromova, Or Aharonov, Zoltán Griger, Ihsane Hmamouchi, Imane El bouchti, Zineb Baba, Margherita Giannini, François Maurier, Julien Campagne, Alain Meyer, Daman Langguth, Vidya Limaye, Merrilee Needham, Nilesh Srivastav, Marie Hudson, Océane Landon-Cardinal, Wilmer Gerardo Rojas Zuleta, Álvaro Arbeláez, Javier Cajas, José António Pereira Silva, João Eurico Fonseca, Olena Zimba, Uyi Ima-Edomwonyi, Ibukunoluwa Dedeke, Emorinken Airenakho, Nwankwo Henry Madu, Abubakar Yerima, Hakeem Olaosebikan, A Becky, Oruma Devi Koussougbo, Elisa Palalane, Ho So, Manuel Francisco Ugarte-Gil, Lyn Chinchay, José Proaño Bernaola, Victorio Pimentel, Hanan Mohammed Fathi, Reem Hamdy A Mohammed, Ghita Harifi, Yurilís Fuentes-Silva, Karoll Cabriza, Jonathan Losanto, Nelly Colaman, Antonio Cachafeiro-Vilar, Generoso Guerra Bautista, Enrique Julio Giraldo Ho, Raúl González, Lilith Stange Nunez, Cristian Vergara M, Jossiell Then Báez, Hugo Alonzo, Carlos Benito Santiago Pastelin, Rodrigo García Salinas, Alejandro Quiñónez Obiols, Nilmo Chávez, Andrea Bran Ordóñez, Sandra Argueta, Gil Alberto Reyes Llerena, Radames Sierra-Zorita, Dina Arrieta, Eduardo Romero Hidalgo, Ricardo Saenz, Idania Escalante M, Roberto Morales, Wendy Calapaqui, Ivonne Quezada, Gabriela Arredondo

**Affiliations:** Department of Precision and Regenerative Medicine and Ionian Area, Rheumatology Unit, University of Bari, Bari, Italy; Department of Precision and Regenerative Medicine and Ionian Area, Rheumatology Unit, University of Bari, Bari, Italy; Department of Clinical Sciences and Community Health, Research Center for Adult and Pediatric Rheumatic Diseases, Università degli Studi di Milano, Milan, Italy; Department of Precision and Regenerative Medicine and Ionian Area, Rheumatology Unit, University of Bari, Bari, Italy; Byramjee Jeejeebhoy Government Medical College and Sassoon General Hospitals, Pune, India; Division of Allergy, Immunology and Rheumatology, Department of Internal Medicine, Taichung Veterans General Hospital, Taichung, Taiwan; Department of Medical Research, Taichung Veterans General Hospital, Taichung, Taiwan; NIHR Leeds Biomedical Research Centre, Leeds Teaching Hospitals Trust, Leeds, UK; Leeds Institute of Rheumatic and Musculoskeletal Medicine, University of Leeds, Leeds, UK; Mymensingh Medical College, Mymensingh, Bangladesh; Department of Internal Medicine, University of Illinois College of Medicine at Peoria, Peoria, IL, USA; Mahatma Gandhi Mission Medical College, Navi Mumbai, Maharashtra, India; Division of Rheumatology, Faculdade de Medicina FMUSP, Universidade de Sao Paulo, Sao Paulo, SP, Brazil; School of Medicine, Universidade Potiguar (UnP), Brazil; Seth Gordhandhas Sunderdas Medical College and King Edwards Memorial Hospital, Mumbai, Maharashtra, India; Rheumatology Department, Saint-Joseph University, Beirut, Lebanon; Rheumatology Department, Hotel-Dieu de France Hospital, Beirut, Lebanon; Medical Faculty, Sofia University St Kliment Ohridski, Sofia, Bulgaria; Department of Rheumatology, Green Life Medical College, Dhaka, Bangladesh; Department of Rheumatology, Clinic of Rheumatology, University Hospital ‘St Ivan Rilski’, Medical University, Sofia, Bulgaria; Department of Internal Medicine, Rheumatology, Diabetology, Geriatrics and Clinical Immunology, Pomeranian Medical University in Szczecin, Szczecin, Poland; Rheumatology Unit, Department of Internal Medicine, Hospital Sultanah Bahiyah, Alor Setar, Malaysia; Department of Internal Medicine, General Hospital, National Medical Center “La Raza”, Instituto Mexicano del Seguro Social, Mexico City, Mexico; Rheumatology Unit, Dipartimento di Medicine Interna e Terapia Medica, Università degli studi di Pavia, Pavia, Lombardy, Italy; National Center for Rheumatic Diseases (NCRD), Ratopul, Kathmandu, Nepal; Department of Allergy and Rheumatology, Nippon Medical School Graduate School of Medicine, Bunkyo-ku, Tokyo, Japan; Faculty of Medicine, Universiti Kebangsaan Malaysia, Cheras, Kuala Lumpur, Malaysia; Medizinische Klinik 3—Rheumatologie und Immunologie, Universitätsklinikum Erlangen, Friedrich-Alexander-Universität Erlangen-Nürnberg, Erlangen, Germany; Division of Rheumatology, Mayo Clinic, Rochester, MN, USA; Rheumatology, Medical Care & Research, Centro Medico Pensiones Hospital, Instituto Mexicano del Seguro Social Delegación Yucatán, Yucatán, Mexico; Rheumatologist and Clinical Investigator, Interstitial Lung Disease and Rheumatology Unit, Instituto Nacional de Enfermedades Respiratorias, Mexico City, Mexico; Department of Internal Medicine, Section of rheumatology, King Faisal Specialist Hospital and Research Center, Jeddah, Saudi Arabia; Rheumatology Unit, Department of Medicine and Therapeutics, University of Ghana Medical School, College of Health Sciences, Korle-Bu, Accra, Ghana; General Director, Reference Center for Osteoporosis, Rheumatology and Dermatology, Pontifica Universidad Javeriana Cali, Colombia; Department of Medicine, Queen Savang Vadhana Memorial Hospital, Chonburi, Thailand; Department of Medicine, Hospital Universidad del Norte, Barranquilla, Atlantico, Colombia; Department of Rheumatology, University Hospital Zurich, University of Zurich, Zurich, Switzerland; Division of Rheumatology, Department of Medicine, Faculty of Medicine Siriraj Hospital, Mahidol University, Bangkok, Thailand; Walter and Eliza Hall Institute of Medical Research, Parkville, VIC, Australia; Department of Medical Biology, University of Melbourne, Parkville, VIC, Australia; Department of Rheumatology, Royal Melbourne Hospital, Parkville, VIC, Australia; Division of Rheumatology, Department of Medicine Solna, Karolinska Institutet and Karolinska University Hospital, Stockholm, Sweden; Department of Rheumatology, Faculty of Medicine and Health, Örebro University, Örebro, Sweden; Centre for Rheumatic Diseases, King’s College London, London, UK; Rheumatology Department, King’s College Hospital, London, UK; Division of Musculoskeletal and Dermatological Sciences, Faculty of Biology, Medicine and Health, Manchester Academic Health Science Centre The University of Manchester, Manchester, UK; Department of Rheumatology, Salford Royal Hospital, Northern Care Alliance NHS Foundation Trust, Salford, UK; Department of Clinical Immunology and Rheumatology, Sanjay Gandhi Postgraduate Institute of Medical Sciences, Lucknow, India; Division of Musculoskeletal and Dermatological Sciences, Faculty of Biology, Medicine and Health, Manchester Academic Health Science Centre The University of Manchester, Manchester, UK; Department of Rheumatology, Royal Wolverhampton Hospitals NHS Trust, Wolverhampton, UK

**Keywords:** comorbidity, autoinflammatory condition, myositis, quality of life, observational studies

## Abstract

**Objective:**

The presence of comorbidities can substantially affect patients’ quality of life, but data regarding their impact on idiopathic inflammatory myopathies (IIMs) are limited.

**Methods:**

We examined the prevalence of comorbidities in IIM patients, other autoimmune rheumatic diseases (oAIRDs) and healthy controls (HCs), using data from the self-reported COVAD-2 survey. We defined basic multimorbidity (BM) as the presence of ≥2 non-rheumatic chronic conditions and complex multimorbidity (CM) as the presence of ≥3 non-rheumatic chronic conditions affecting ≥3 organ systems. Hierarchical clustering on principal components was performed for grouping.

**Results:**

Among the COVAD respondents, 1558 IIMs, 4591 oAIRDs and 3652 HCs were analysed. IIMs exhibited a high burden of comorbidities (odds ratio [OR]: 1.62 *vs* oAIRDs and 2.95 *vs* HCs, *P* < 0.01), BM (OR: 1.66 *vs* oAIRDs and 3.52 *vs* HCs, *P* < 0.01), CM (OR: 1.69 *vs* AIRDs and 6.23 *vs* HCs, *P* < 0.01) and mental health disorders (MHDs) (OR: 1.33 *vs* oAIRDs and 2.63 *vs* HCs, *P* *<* 0.01). Among the IIM patients, those with comorbidities or MHDs had lower PROMIS Global Physical (PGP), PROMIS Global Mental (PGM), and PROMIS Physical Function (SF10) scores, and higher fatigue (F4a) scores (all *P* *<* 0.001). PGP, PGM, SF10a and F4a were influenced by age, active disease, BM and MHDs. Four distinct clusters were identified among the IIMs according to comorbidities and PROMIS scores.

**Conclusion:**

Patients with IIMs have a higher burden of comorbidities that influence physical and mental health, identifiable as clinical clusters for optimized and holistic management approaches.

Rheumatology key messages

- Among the idiopathic inflammatory myopathies patients, those with comorbidities or mental health disorders had worse physical function.
- Worse physical and mental health were predicted by age, active disease, basic multimorbidity and mental health disorders.
- Four distinct clusters were identified among the idiopathic inflammatory myopathies according to comorbidities and PROMIS scores.

## Introduction

Idiopathic inflammatory myopathies (IIMs) are chronic, multi-system disorders that often require long-term treatment with corticosteroids and immunosuppressants [1]. These conditions frequently affect the elderly, who are more vulnerable to comorbidities [2–4]. These co-existing health issues can complicate the management of IIM and adversely affect quality of life (QoL) [5–7].

Chronic illnesses, by nature, often lead to secondary health issues, either as a direct result of the illness or from treatment interventions [8]. This complexity, marked by multiple chronic conditions, demands comprehensive healthcare and can lead to a high burden of diagnostic assessments and fragmented specialized care. The interconnection of these challenges highlights the importance of a collaborative, interdisciplinary approach to ensure best outcomes for patients [9].

Modern social changes, including longer lifespans and widening socioeconomic gaps, contribute to an increasing trend in prevalence of comorbidities [10, 11]. While comorbidity in some autoimmune disorders like rheumatoid arthritis (RA) [12, 13] and systemic lupus erythematosus (SLE) [14] has been well-studied, there is a notable scarcity of data on IIMs.

Using data from the COVAD-2 database, this study explored the prevalence of multimorbidity in patients with IIMs compared with other autoimmune rheumatic diseases (oAIRDs) and healthy controls (HCs). Additionally, it examined the impact of comorbidities on patients' physical, mental and social health using the Patient-Reported Outcome Measurement Information System (PROMIS) instruments.

## Methods

### Data source

The study utilized the COVAD-2 dataset, generated by the COVAD study group through a patient-reported global e-survey, which was extensively pilot tested, validated and translated into 18 languages. The methodology has been previously published [15]. The COVAD study group included 157 physicians across 106 countries, collecting data from patient cohorts in clinics or via patient support groups [15].

### The COVAD dataset, information extraction and cleaning

This study was designed to assess patient demographics, underlying autoimmune condition and associated characteristics such as disease duration, comorbidities, current treatment and flares, patient-reported QoL, fatigue and pain, country and ethnicity, and COVID-19 vaccination and infections. The data were collected between January and August 2022, extracted in August 2022, and the responses were screened. Duplicate or incomplete responses were excluded.

Data variables were categorized for assessment, with special emphasis on comorbidities. Patients who reported a diagnosis made by a rheumatologist or a physician experienced in rheumatic diseases were included. Further data cleaning was undertaken by categorizing those with multiple diagnoses by the most likely one—based on symptoms described. To avoid erroneous duplicated and incomplete entries, we removed incomplete entries and those that lacked a clear diagnosis.

### Study population

The population was divided into three groups: patients’ IIM, including dermatomyositis (DM), polymyositis (PM), inclusion body myositis (IBM), immune-mediated necrotizing myopathy (IMNM), anti-synthetase syndrome disease (ASyS) and overlap myositis (OM); patients with oAIRDs (inflammatory arthritis: RA, spondyloarthritis; connective tissue diseases [CTDs]: SLE, Sjögren’s syndrome, systemic sclerosis and systemic vasculitis), and a control group with healthy individuals (HCs). OM was defined by the coexistence of a diagnosis of IIM and CTD in the same individual. Patients diagnosed with non-rheumatic autoimmune diseases were excluded.

### Ethical approval

Ethical approval was obtained from the Institutional Ethics Committee of SGPGIMS (Lucknow, India).

### Comorbidity assessment

The COVAD dataset extensively recorded comorbidities and mental health disorders (MHD). The comorbidities recorded are detailed in Supplementary Table S1, available in at *Rheumatology* online. Multimorbidity was defined as having more than one comorbidity beyond a rheumatic disease. These were further classified into basic multimorbidity (BM), defined as the presence of two or more chronic diseases from the aforementioned list, and complex multimorbidity (CM), defined as the presence of three or more chronic conditions affecting three or more different body systems [11] (Supplementary Table S1, available in at *Rheumatology* online).

### Patient outcomes

Demographic data and the Human Development Index (HDI) of each subject's country of origin were collected, with HDI assessing a country's development through life expectancy, education and income indicators. PROMIS scores, including global physical health (PGP), global mental health (PGM), fatigue 4a (F4a), and physical function (SF10), were also acquired (Supplementary Table S2, available in at *Rheumatology* online). Among IIMs, disease activity was determined by patients’ perception of the disease, assessed by a specific question in the survey. Patients who regarded disease activity as inactive or in remission, as well as stable and manageable, were categorized as being in an inactive status. Conversely, patients whose disease activity was described as active and improving, or active and worsening were classified as being in an active status.

### Statistical analysis

Continuous variables were reported as the mean and standard deviation (SD) for normally distributed variables and as the median and interquartile range (IQR) for non-normally distributed variables, whereas categorical variables were expressed as numbers and the corresponding percentage. The Kolmogorov–Smirnov test was used for assessment of normality.

Comparisons between groups were performed by using the chi-square test, Fisher’s exact test, Student’s *t*-test and the Mann–Whitney *U*-test, as appropriate. A logistic regression analysis adjusting for age, sex, ethnicity and HDI value was used to compare the risk of comorbidities in IIMs *vs* comparators (oAIRD and HCs) and among IIM subsets to address the lack of matching features between the cohorts.

Linear regression models were used to investigate the relationship between comorbidities and PROMIS scores (PGP, PGM, F4a and SF10) in the IIM groups. Statistical significance was set at *P* < 0.05. Analyses were performed using SPSS Statistics (version 21; IBM Corp., Armonk, NY, USA).

Hierarchical clustering on principal components was performed to delineate the grouping of IIM patients. Factor analysis with mixed data (FAMD) is a generalization of principal component analysis based on unsupervised machine learning algorithms that can be deployed on large datasets with qualitative and quantitative variables [16]. FAMD was implemented in a Python v. 3.9 environment, using Prince v. 0.7.1, scipy v. 1.9.3, scikit-learn v. 1.1.3 and numpy v. 1.23.2.

Accurate preprocessing with feature standardization and autocorrelation was performed for the continuous variables. Observations with missing data were ablated. One hundred repeats with different seeds were carried out, and the 95% confidence interval (CI) of the bootstrapped eigenvalue distribution was used to determine the number of FAMD components retained for clustering. To define the number of clusters, hierarchical clustering on principal components was performed using Ward’s criterion and a Euclidian distance metric on the selected FAMD principal components [17]. The inter-cluster inertia gain was used to determine the optimal division level.

## Results

### Characteristics of study participants

Out of 10 740 respondents, 398 were excluded for incomplete data, and 541 for having non-rheumatic autoimmune diseases, resulting in a final cohort of 1558 individuals with IIMs, 4591 with oAIRDs and 3652 HCs (Supplementary Fig. S1, available at *Rheumatology* online). The IIM group included 470 (30.2%) with DM, 217 (13.9%) with PM, 375 (24.1%) with IBM, 103 (6.6%) with ASyS, 96 (6.2%) with IMNM, and 239 (15.3%) with OM. The oAIRDs group comprised 2147 (46.8%) with inflammatory arthritis, 2123 (44.2%) with CTDs, and 321 (7%) with systemic vasculitis.

IIM patients were generally older (median [IQR]: 61 [49–70] years) compared with oAIRDs (48 [37–59] years) and HCs (38 [30–49] years). The cohort was predominantly female: 71.4% in IIMs, 84% in oAIRDs, and 60.2% in HCs. Most were Caucasian (77.5% in IIMs, 45.9% in oAIRDs and 28.9% in HCs). Respondents with rheumatic conditions were primarily from countries with a very high HDI: 93.1% in IIMs, 60.8% in oAIRDs and 29.9% in HCs. Demographic data are detailed in Table 1.

**Table 1.** Demographic characteristics of overall cohort

	IIMs (*n* = 1558)	oAIRDs (*n* = 4591)	HCs (*n* = 3652)	*P*-value
Age, median (IQR), years	61 (49–70)	48 (37–59)	38 (30–49)	*<*0.001
Gender, *n* (%)				*<*0.001
Male	462 (27.3)	677 (14.7)	1390 (38.1)	
Female	1113 (71.4)	3856 (84)	2200 (60.2)	
Others	9 (0.6)	32 (0.7)	28 (0.8)	
Ethnicity, *n* (%)				*<*0.001
Caucasian (White)	1207 (77.5)	2105 (45.9)	1057 (28.9)	
Asian	125 (8)	1066 (23.2)	909 (24.9)	
African American	64 (4.1)	297 (6.5)	188 (5.1)	
Hispanic	66 (4.2)	465 (10.1)	889 (24.3)	
Mixed	31 (2)	174 (3.8)	252 (6.9)	
Native American	4 (0.3)	44 (1.0)	29 (0.8)	
Do not wish to disclose	30 (1.9)	171 (3.7)	162 (4.4)	
Others	21 (1.3)	243 (5.3)	132 (3.6)	
HDI, *n* (%)				*<*0.001
Very high	1451 (93.1)	2791 (60.8)	1093 (29.9)	
High	68 (4.4)	1035 (22.5)	1354 (37.1)	
Medium	23 (1.5)	608 (13.2)	955 (26.2)	
Low	6 (0.4)	128 (2.8)	215 (5.9)	

HCs: healthy controls; HDI: Human Development Index; IIMs: idiopathic inflammatory myopathies; oAIRDs: other autoimmune rheumatic diseases.

### Multimorbidity

Comorbidities were common among patients with rheumatic diseases, present in 67.4% of IIM patients, 44.1% of oAIRD patients and 25% of HCs. IIM patients had a higher likelihood of comorbidities compared with oAIRD patients (odds ratio [OR]: 1.62, 95% CI: 1.42, 1.86) and HCs (OR: 2.95, 95% CI: 2.45, 3.53) (Table 2).

**Table 2.** Distribution of comorbidities in IIMs, oAIRDs and HCs

				OR (95% CI)^a^	
	IIMs (*n* = 1558)	oAIRDs (*n* = 4591)	HCs (*n* = 3652)	IIMs *vs* oAIRDs	IIMs *vs* HCs
Comorbidities overall, *n* (%)	1050 (67.4)	2023 (44.1)	914 (25)	1.62 (1.42, 1.86)	2.95 (2.45, 3.53)
Basic multimorbidity, *n* (%)	545 (35)	745 (16.2)	226 (6.2)	1.66 (1.43, 1.93)	3.52 (2.72, 5.54)
Complex multimorbidity, *n* (%)	218 (14)	297 (6.5)	50 (1.4)	1.69 (1.37, 2.08)	6.23 (3.98, 9.74)
Mental health disorders, *n* (%)	530 (34)	1289 (28.1)	632 (17.3)	1.33 (1.16, 1.52)	2.63 (2.15, 3.22)
List of comorbidities, *n* (%)					
Asthma	227 (14.6)	508 (11.1)	256 (7)	ns	1.82 (1.39, 2.38)
CKD	56 (3.6)	243 (5.3)	22 (0.6)	0.58 (0.42, 0.81)	5.51 (2.58, 11.75)
CLD	29 (1.9)	63 (1.4)	21 (0.6)	ns	ns
COPD	62 (4)	131 (2.9)	32 (0.9)	ns	2.72 (1.34, 5.54)
ILD	265 (17)	156 (3.4)	9 (0.2)	5.1 (4.03, 6.45)	53.02 (24.64, 114.07)
IHD	123 (7.9)	117 (2.5)	33 (0.9)	1.63 (1.2, 2.2)	3.32 (1.83, 6.05)
DM	208 (13.4)	259 (5.6)	148 (4.1)	1.71 (1.36, 2.14)	2.45 (1.05, 3.49)
Epilepsy	9 (0.6)	64 (1.4)	10 (0.3)	ns	ns
Dyslipidaemia	384 (24.6)	585 (12.7)	265 (7.3)	1.38 (1.17, 1.63)	1.78 (1.38, 2.29)
HIV-AIDS	8 (0.5)	7 (0.2)	10 (0.3)	4.12 (1.16, 14.61)	ns
Hypertension	503 (32.2)	899 (19.6)	370 (10.1)	1.21 (1.04, 1.40)	1.87 (1.47, 2.39)
Stroke	27 (1.7)	47 (1)	9 (0.2)	ns	ns
Tuberculosis	8 (0.5)	55 (1.2)	16 (0.4)	ns	ns
Organ transplant	4 (0.3)	19 (0.4)	7 (0.2)	ns	ns
Others	126 (8.1)	198 (4.3)	51 (1.4)	ns	1.69 (1.07, 2.69)
List of mental health disorders, *n* (%)					
Anxiety	329 (21.1)	783 (17.1)	417 (11.4)	1.37 (1.17, 1.62)	2.69 (2.12, 3.43)
Bipolar disorder	16 (1)	30 (0.7)	21 (0.6)	2.28 (1.14, 4.56)	3.04 (1.18, 7.79)
Depression	307 (19.7)	698 (15.2)	285 (7.8)	1.32 (1.12, 1.56)	3.63 (2.78, 4.72)
Eating disorder	21 (1.3)	98 (2.1)	48 (1.3)	ns	ns
Insomnia	138 (8.9)	362 (7.9)	113 (3.1)	1.34 (1.07, 1.69)	3.95 (2.64, 5.92)
Schizophrenia	3 (0.2)	14 (0.3)	3 (0.1)	ns	ns
Substance use disorders	6 (0.4)	19 0.4)	5 (0.1)	ns	ns

a Logistic analysis adjusted for age, gender, ethnicity, Human Development Index value. CLD: chronic liver disease; CKD: chronic kidney disease; COPD: chronic obstructive pulmonary disease; DM: diabetes mellitus; HCs: healthy controls; HIV-AIDS: human immunodeficiency virus-acquired immune deficiency syndrome; IHD: ischaemic heart disease; IIMs: idiopathic inflammatory myopathies; ILD: interstitial lung disease; ns: not significant; oAIRDs: other autoimmune rheumatic diseases; OR: odds ratio.

BM (at least two comorbidities) occurred in 35% of IIM patients, 16.2% of oAIRD patients and 6.2% of HCs, with IIM patients at greater risk than oAIRD patients (OR: 1.66, 95% CI: 1.43, 1.93) and HCs (OR: 3.52, 95% CI: 2.72, 5.54).

CM was seen in 14% of IIM patients, 6.5% of oAIRD patients and 1.4% of HCs, with IIM patients having a higher risk than oAIRD patients (OR: 1.69, 95% CI: 1.37, 2.08) and HCs (OR: 6.23, 95% CI: 3.98, 9.74).

MHDs were present in 34% of IIM patients, 28.1% of oAIRD patients and 17.3% of HCs, with IIM patients more likely to have MHDs compared with oAIRD patients (OR: 1.33, 95% CI: 1.16, 1.52) and HCs (OR: 2.63, 95% CI: 2.15, 3.22).

Considering the origin of patients from different continents, we performed a subanalysis by continent, considering Europe (218 IIMs, 1754 oAIRDs and 668 HCs), North America (1028 IIMs, 650 oAIRDs and 945 HCs), Asia (135 IIMs, 1320 oAIRDs and 1151 HCs) and the remaining continents (Australia, South America, Africa: 177 IIMs, 867 oAIRDs and 888 HCs). The analysis confirmed that patients with IIMs had a higher risk of at least one comorbidity, BM, CM and MHDs compared with HCs. Compared with oAIRDs, patients with IIMs were confirmed to have a higher risk for most definitions of comorbidities in Europe, Asia and the remaining continents, while in North America, there was a trend towards a higher risk in patients with IIMs (data shown in Supplementary Table S3, available in at *Rheumatology* online).

The full list of comorbidities in IIMs, AIRDs and HCs is reported in Table 2.

In IIM patients compared with oAIRDs, there was a higher risk of interstitial lung disease (ILD) (OR: 5.1), ischaemic heart disease (IHD) (OR: 1.63), diabetes mellitus (OR: 1.71), dyslipidaemia (OR: 1.38), HIV-AIDS (OR: 4.12) and arterial hypertension (OR: 1.21). For mental health disorders (MHDs), IIM patients had higher risks of anxiety (OR: 1.37), bipolar disorder (OR: 2.28), depression (OR: 1.32) and insomnia (OR: 1.34). However, IIM patients had a lower risk of chronic kidney disease (CKD) (OR: 0.58) compared with oAIRDs.

When comparing IIM patients with HCs, they had higher risks of asthma (OR: 1.82), CKD (OR: 5.51), chronic obstructive pulmonary disease (OR: 2.72), ILD (OR: 53.02), IHD (OR: 3.32), diabetes mellitus (OR: 2.45), dyslipidaemia (OR: 1.78) and arterial hypertension (OR: 1.87). For MHDs, IIM patients showed significantly increased risks of anxiety (OR: 2.69), bipolar disorder (OR: 3.04), depression (OR: 3.63) and insomnia (OR: 3.95) compared with HCs.

Considering that ILD may be a possible manifestation related to AIRDs, we performed the same analysis excluding ILD from the count of comorbidities in IIMs and oAIRDs. No variations were observed, as IIMs continued to show a higher burden of comorbidities, BM and CM compared with both oAIRDs and HCs (Supplementary Table S4, available in at *Rheumatology* online).

### Multimorbidity in subsets of IIM

Supplementary Fig. S2, available at *Rheumatology* online, shows the densities of BM, CM and MHDs in patients with IIMs according to their different geographic areas of origin. While the burden of BM (Supplementary Fig. S2A, available at *Rheumatology* online) and CM (Supplementary Fig. S2B, available at *Rheumatology* online) appears higher in certain areas, such as North America and Europe, it is evident that MHDs are consistently observed across multiple regions of the world (Supplementary Fig. S2C, available at *Rheumatology* online).

Table 3 shows the comorbidities in the different IIM subsets. According to logistic regression analysis, patients with PM and ASyS had a higher risk of having at least one comorbidity (OR: 2.19, 95% CI: 1.21, 3.19; OR: 2.57, 95% CI: 1.53, 4.34, respectively) compared with other subsets of IIM, whereas patients with DM had a lower risk (OR: 0.70, 95% CI: 0.54, 0.89), which persisted despite adjusting for age, sex, ethnicity and country HDI. The presence of BM (at least two comorbidities) was more common in patients with PM (OR: 1.68, 95% CI: 1.24, 2.29), ASyS (OR: 1.56, 95% CI: 1.01, 2.42), and IMNM (OR: 1.72, 95% CI: 1.12, 2.67), while a lower likelihood was observed for patients with DM (OR: 0.64, 95% CI: 0.50, 0.82), and IBM (OR: 0.73, 95% CI: 0.54, 0.97).

**Table 3.** Characteristics of the respondents in the cohort in IIMs

	DM (*n* = 470)	PM (*n* = 217)	IBM (*n* = 375)	ASS (*n* = 103)	IMNM (*n* = 96)	OM (*n* = 239)
Age, median (IQR), years	58 (48–67)	64 (50–70)	71 (65–76)	52 (42–63)	57 (49–66)	49 (40–60)
Gender, *n* (%)						
Male	84 (17.9)	54 (24.9)	221 (58.9)	16 (15.5)	27 (28.1)	18 (7.5)
Female	383 (81.5)	161 (74.2)	152 (40.5)	85 (82.5)	69 (71.9)	213 (89.1)
Do not wish to disclose	1 (0.2)	1 (0.4)	2 (0.5)	1 (0.2)	0 (0)	1 (0.4)
Others	1 (0.2)	1 (0.4)	0 (0)	0 (0)	0 (0)	1 (0.4)
Ethnicity, *n* (%)						
Caucasian (White)	378 (80.4)	171 (78.8)	349 (93.1)	81 (78.6)	74 (77.1)	120 (50.2)
Asian	24 (5.1)	13 (6)	6 (1.6)	9 (8.7)	7 (7.3)	58 (24.3)
African American	20 (4.3)	13 (6)	4 (1.1)	2 (1.9)	6 (6.3)	13 (5.4)
Hispanic	31 (6.6)	5 (2.3)	9 (2.4)	3 (2.9)	7 (7.3)	8 (3.3)
Mixed	10 (2.1)	5 (2.3)	0 (0)	4 (3.9)	1 (1)	9 (3.8)
Native American	0 (0)	0 (0)	1 (0.3)	0 (0)	0 (0)	2 (0.8)
Do not wish to disclose	6 (1.3)	4 (1.8)	5 (1.3)	0 (0)	1 (1)	13 (5.4)
Others	0 (0)	4 (1.8)	5 (1.3)	0 (0)	1 (1)	13 (5.4)
HDI, *n* (%)						
Very high	453 (96.4)	202 (93.1)	370 (98.7)	100 (97.1)	93 (96.9)	187 (78.2)
High	12 (2.6)	7 (3.2)	4 (1.1)	2 (1.9)	3 (3.1)	35 (14.6)
Medium	4 (0.9)	5 (2.3)	1 (0.3)	0 (0)	0 (0)	6 (2.5)
Low	0 (0)	1 (0.5)	0 (0)	0 (0)	0 (0)	5 (2.1)
Disease activity, *n* (%)						
Inactive	328 (88.2)	137 (82.5)	142 (49)	64 (81)	50 (72.5)	125 (86.8)
Active	44 (11.8)	29 (17.5)	148 (51)	15 (19)	19 (27.5)	19 (13.2)
Comorbidities overall, *n* (%)	285 (60.6)	174 (80.2)	269 (71.7)	83 (80.6)	66 (68.6)	133 (55.6)
OR (95% CI)	**0.7 (0.54, 0.89)**	**2.19 (1.21, 3.19)**	ns	**2.57 (1.53, 4.34)**	ns	ns
Basic multimorbidity, *n* (%)	127 (27)	100 (46.1)	153 (40.8)	40 (38.8)	43 (44.8)	65 (27.2)
OR (95% CI)	**0.64 (0.5, 0.82)**	**1.68 (1.24, 2.29)**	**0.73 (0.54, 0.97)**	**1.56 (1.01, 2.42)**	**1.72 (1.12, 2.67)**	ns
Complex multimorbidity, *n* (%)	50 (10.6)	39 (18)	44 (11.7)	16 (15.5)	23 (24)	35 (14.6)
OR (95% CI)	**0.6 (0.42, 0.85)**	ns	ns	ns	**2.05 (1.23, 3.39)**	ns
Mental health disorders, *n* (%)	166 (35.3)	76 (35)	105 (28)	31 (30.1)	34 (35.4)	94 (39.3)
OR (95% CI)	ns	ns	ns	**0.61 (0.38, 0.96)**	ns	ns

Logistic analysis (bold) adjusted for age, gender, ethnicity, HDI value is reported as OR (95% CI). ASS: antisynthetase syndrome; DM: dermatomyositis; HDI: Human Development Index; IBM: inclusion body myositis; IMNM: immune mediated necrotizing myopathy; ns: not significantly different from other idhiopatic inflammatory myopathies; OM: overlap myositis; PM: polymyositis; OR: odds ratio.

Notably, individuals with IMNM were more likely to be diagnosed with concomitant CM, (OR: 2.05, 95% CI: 1.23, 3.39), whereas a lower probability was observed for patients with DM (OR: 0.60, 95% CI: 0.42, 0.85) compared with other subsets of IIM.

Finally, MHDs were evenly distributed across all IIMs subsets, except for ASyS, where a reduced risk was observed compared with other IIM subsets (OR: 0.61, 95% CI: 0.38, 0.96). A complete list of comorbidities in patients with IIMs is provided in Supplementary Table S5, available in at *Rheumatology* online. Additionally, we conducted the analysis excluding ILD as a comorbidity in IIMs, and the results are reported in Supplementary Table S6, available in at *Rheumatology* online. In this case, an inversion of the risk of having at least one comorbidity for ASS was observed (OR: 0.64, 95% CI: 0.42, 0.99), while no increased risk was observed for BM and CM.

### PROMIS outcome measures in IIMs

Table 4 reports the PROMIS results in IIM patients. As observed, IIM patients with BM, CM and MHDs had worse physical function, including low PGP, PGM and SF10, and higher F4a scores compared with patients without.

**Table 4.** PROMIS outcome measures among IIMs according to comorbidities

	Basic multimorbidity		Complex multimorbidity		MHDs	
	Yes	No	Yes	No	Yes	No
PROMIS Global Physical health score	11 (9–14)	13 (11–15)*	11 (9–13)	12 (10–15)*	11 (9–13)	13 (11–15)*
PROMIS Global Mental Health score	12 (10–15)	13 (11–16)*	12 (9–14)	13 (11–16)*	11 (9–14)	14 (11–16)*
PROMIS Physical function SF10a score	31 (23–37)	37 (27–44.5)*	30 (23–36.25)	35 (26–43)*	32 (25–40)	54 (26–44)*
PROMIS Fatigue 4a score (0–20)	12 (8–16)	11 (8–14)*	13 (10–16)	11 (8–14)*	13 (10–16)	10 (8–13)*
Pain VAS of PROMIS (0–10)	3 (1–5)	3 (1–5)*	4 (2–6)	3 (1–5)*	4 (2–6)	2 (0–4)*
Fatigue VAS of PROMIS (1–5)	3 (2–3)	3 (2–3)*	3 (3–4)	3 (2–3)*	3 (3–4)	3 (2–3)*

* *P* *<* 0.001. MHD: mental health disorders; PROMIS: Patient-Reported Outcome Measurement Information System; VAS: visual analogue scale.

Among the IIMs, PGP was positively correlated with age (regression coefficient [*B*]: 0.35; *P* = 0.030) and negatively correlated with active disease (*B*: −1.51; *P* < 0.001), BM (*B*: −1.11; *P* < 0.001) and MHDs (*B*: −1.47; *P* < 0.001). PGM was positively correlated with age (*B*: 0.51; *P* = 0.004) and negatively correlated with active disease (*B*: −1.34, *P* < 0.001), BM (*B*: −0.75; *P* = 0.001) and MHDs (*B*: −2.22; *P* < 0.001). SF10a included a negative correlation with age (*B*: −3.86; *P* < 0.001), active disease (*B*: −7.03; *P* < 0.001), BM (*B*: −2.95; *P* < 0.001) and MHDs (*B*: −2.37; *P* < 0.001), but female gender was positively correlated (*B*: 2.85; *P* < 0.001). F4a score was negatively correlated with age (*B*: −0.96, *P* < 0.001) and showed a positive correlation with active disease (*B*: 1.45, *P* < 0.001), country HDI (*B*: 0.95; *P* = 0.04), BM (*B*: 1.11; *P* < 0.001) and MHDs (*B*: 2.17; *P* < 0.001) (Table 5).

**Table 5.** Linear regression analysis of predictors of PROMIS outcomes in IIMs

	PROMIS Global Physical Health		PROMIS Global mental health		PROMIS Physical function (SF10 score)		Fatigue 4a	
	*B*	*P*-value	*B*	*P*-value	*B*	*P*-value	*B*	*P*-value
Age	0.35	0.03	0.51	0.004	−3.86	<0.001	−0.96	<0.001
Active disease	−1.51	<0.001	−1.34	<0.001	−7.03	<0.001	1.45	<0.001
Gender (F/M)	0.08	0.67	−0.12	0.59	2.85	<0.001	0.33	0.21
Basic multimorbidity	−1.12	<0.001	−0.75	0.001	−2.95	<0.001	1.11	<0.001
Complex multimorbidity	0.27	0.39	0.40	0.26	−0.01	1.00	−0.24	0.58
Mental health disorders	−1.47	<0.001	−2.22	<0.001	−2.36	0.001	2.17	<0.001
HDI classification	−0.56	0.09	−0.11	0.76	−0.04	0.97	0.95	0.04

*B*: regression coefficient; F: female; HDI: Human Development Index; M: male; PROMIS: Patient-Reported Outcome Measurement Information System.

### Cluster analysis

Hierarchical clustering on principal components identified four distinct clusters of IIM patients (Table 6). Cluster 1 included younger patients with fewer comorbidities and good health. Cluster 2 comprised older patients with a higher prevalence of CM, MHDs, metabolic syndrome-related comorbidities and a higher burden of cardiovascular events. Cluster 3 featured a higher prevalence of MHDs, low PGP/PGM scores and a high F4a score, with common conditions like depression, anxiety and insomnia. Cluster 4, consisting of older patients with higher BM but better health as indicated by PROMIS scores, showed less comorbidity impact than cluster 2.

**Table 6.** Cluster analysis in idiopathic inflammatory myopathies

	Cluster 1 (*n* = 219)	Cluster 2 (*n* = 564)	Cluster 3 (*n* = 318)	Cluster 4 (*n* = 422)
Female, *n* (%)	189 (86.3)^a^	358 (63.5)^b^	288 (90.6)^a^	258 (61.1)^b^
Age, mean (SD)	44 (13)^a^	66 (10)^b^	45 (12)^a^	66 (9)^b^
BM, *n* (%)	10 (4.6)^a^	330 (58.5)^b^	44 (13.8)^c^	156 (37)^d^
CM, *n* (%)	4 (1.8)^a^	153 (27.1)^b^	27 (8.5)^c^	31 (7.3)^c^
Ethnicity, *n* (%)				
Caucasian (White)	115 (52.5)^a^	502 (89)^b^	192 (60.4)^a^	386 (91.5)^b^
Asian	59 (26.9)^a^	2 (0.4)^b^	52 (16.4)^c^	8 (1.9)^b^
African American	10 (4.6)^a^	26 (4.6)^a^	15 (4.7)^a^	12 (2.8)^a^
Hispanic	13 (5.9)^a, b^	15 (2.7)^b, c^	28 (8.8)^a^	7 (1.7)^c^
Very high HDI, *n* (%)	176 (80.4)^a^	557 (98.8)^b^	277 (87.1)^a^	419 (99.3)^b^
Asthma	9 (4.1)^a^	119 (21.1)^b^	50 (15.7)^b, c^	46 (10.9)^c^
CKD	3 (1.4)^a^	28 (5)^a^	12 (3.8)^a^	11 (2.6)^a^
CLD	2 (0.9)^a^	16 (2.8)^a^	5 (1.6)^a^	5 (1.2)^a^
COPD	3 (1.4)^a^	44 (7.8)^b^	7 (2.2)^a^	8 (1.9)^a^
ILD	40 (18.3)^a^	93 (16.5)^a^	55 (17.3)^a^	73 (17.3)^a^
IHD	0 (0)^a^	88 (15.6)^b^	7 (2.2)^a^	27 (6.4)^c^
DM	5 (2.3)^a^	130 (23)^b^	13 (4.1)^a^	59 (14)^c^
Epilepsy	0 (0)^a^	6 (1.1)^a^	3 (0.9)^a^	0 (0)^a^
Dyslipidaemia	9 (4.1)^a^	220 (39)^b^	27 (8.5)^a^	123 (29.1)^c^
HIV-AIDS	1 (0.5)^a^	5 (0.9)^a^	1 (0.3)^a^	1 (0.2)^a^
Hypertension	12 (5.5)^a^	289 (51.2)^b^	34 (10.7)^a^	164 (38.9)^c^
Stroke	0 (0)^a^	19 (3.4)^b^	1 (0.3)^a^	7 (1.7)^a,b^
Tuberculosis	0 (0)^a^	5 (0.9)^a^	3 (0.9)^a^	0 (0)^a^
Organ transplant	1 (0.5)	2 (0.4)	0 (0)	0 (0)
Others	2 (0.9)^a^	70 (12.4)^b^	9 (2.8)^a^	43 (10.2)^b^
MHDs, *n* (%)	39 (17.8)^a^	241 (42.7)^b^	176 (55.3)^c^	61 (14.5)^a^
Anxiety	25 (11.4)^a^	146 (25.9)^b^	117 (36.8)^c^	34 (8.1)^a^
Bipolar disorder	1 (0.5)^a,b^	6 (1.1)^a,b^	8 (2.5)^b^	1 (0.2)^a^
Depression	15 (6.8)^a^	153 (27.1)^b^	108 (34)^b^	28 (6.6)^a^
Eating disorder	5 (2.3)^a, b^	4 (0.7)^b,c^	11 (3.5)^a^	0 (0)^a^
Insomnia	7 (3.2)^a^	63 (11.2)^b^	53 (16.7)^b^	10 (2.4)^a^
Schizophrenia	1 (0.5)^a^	1 (0.2)^a^	1 (0.3)^a^	0 (0)^a^
Substance use disorders	0 (0)^a^	2 (0.4)^a^	3 (0.9)^a^	0 (0)^a^
PROMIS Global Physical Health, mean (s.d.)	15.8 (2.1)^a^	10.2 (1.9)^b^	10.5 (2.3)^b^	14.5 (1.7)^c^
PROMIS Global Mental Health, mean (s.d.)	15.4 (2.7)^a^	11.2 (2.7)^b^	10.8 (3.0)^b^	15.2 (2.4)^a^
PROMIS Physical Function SF10a, mean (s.d.)	45.8 (4.5)^a^	25.6 (8.5)^b^	34.3 (8.6)^c^	36.4 (9.5)^d^
PROMIS Fatigue 4a score, mean (s.d.)	7.2 (2.4)^a^	13.7 (3.2)^b^	13.8 (3.3)^b^	8.0 (2.3)^c^

Each superscript letter indicates a subset of the four groups analysed for which the means or proportions showed no difference at a significance level of 0.05. BM: basic multimorbidity; CM: complex multimorbidity; HDI: Human Development Index; MHD: mental health disorder; PROMIS: Patient-Reported Outcome Measurement Information System.

Supplementary Fig. S3, available at *Rheumatology* online, shows the distribution of IIMs across clusters. DM and ASyS were evenly distributed, while IMNM was less common in cluster 1 (11.8%). IBM and PM were more prevalent in clusters 2 (61.7% and 45.3%) and 4 (36.4% and 34.6%), and OM was more common in cluster 3 (45.4%).

## Discussion

To provide holistic, high-quality care for rheumatic patients, understanding the impact of comorbidities is essential. However, only a few studies have assessed this significant impact in rheumatic patients to date. Our analysis of the COVAD cohort shows that IIM patients have a significantly higher burden of comorbidities, including MHDs, compared with both oAIRDs and HCs.

The concept of comorbidity is broad, encompassing the simultaneous presence of multiple conditions within an individual. Recently, the term ‘complex multimorbidity’ has emerged, defined as the presence of three or more chronic conditions affecting three or more different body systems. The CM assessment approach is based on evidence that the occurrence of diseases in multiple body systems can have a significant impact on the overall assessment of health [10]. When applying these rigorous definitions to the COVAD cohort, it becomes evident that IIM patients carry a higher burden of CM, emphasizing the extraordinary complexity that rheumatologists managing these patients must contend with. This further underscores the heightened vulnerability of these patients and reinforces the imperative for a comprehensive approach to their care, addressing not only the underlying rheumatic condition, but also all other facets of their health.

Our study found that IIM patients have a higher risk of IHD, likely due to increased prevalence of risk factors like dyslipidaemia and hypertension. This aligns with other studies [18] showing an independently increased cardiovascular risk in IIM patients [19, 20]. Certainly further research is needed to explore the roles of disease, comorbidities and therapies in this risk.

A significant finding is the higher incidence of MHDs in IIM, compared with oAIRDs and HCs. Our data show that MHDs are a global concern for IIM patients, with a uniform distribution across all regions in the COVAD cohort. This may be due to IIMs being rare diseases with limited access to effective therapies and a high risk of disability, increasing vulnerability to MHDs like anxiety and depression.

Additionally, the use of glucocorticoids, which are commonly prescribed for managing IIMs, can further exacerbate this risk by contributing to the development of MHDs [21].

The PROMIS is a crucial tool for assessing patient health and performs well in IIM patients [22, 23]. In this study, comorbidities—including BM, CM, and MHDs—were linked to worse patient-reported outcomes across multiple health domains, such as global physical and mental health, physical function, and fatigue. Notably, having at least two comorbidities or MHDs was the primary factor negatively impacting all PROMIS domains.

Note that other intriguing correlations were found. Older age was linked to better mental and physical health and less fatigue. Mental health issues like anxiety and depression are common in younger patients with chronic rheumatic diseases [24, 25] and may significantly impact their fatigue levels [26]. Female gender was associated with better physical function, possibly due to the higher prevalence of IBM in males, a condition causing severe physical disability [23]. A higher HDI was linked to greater fatigue, though this finding should be cautiously interpreted due to the small number of IIMs in other HDI categories. Further research is needed to explore this discrepancy.

This approach emphasizes the need for individualized care for these patients, potentially through specialized clinics or prioritized follow-ups to prevent delays, while acknowledging the greater burden on healthcare systems. IIMs represent a diverse group of conditions, involving multiple organ systems and requiring different therapeutic strategies [1]. Our study analysed the distribution of IIM subsets across four identified clusters. DM, ASyS and IMNM were evenly distributed, whereas IBM and PM were primarily found in clusters 2 and 4. Notably, most IBM and PM patients were in cluster 2, characterized by high comorbidity prevalence and poorer PROMIS outcomes. IBM, common in older adults, often leads to profound disability and significantly impacts patient-reported outcome scores [23, 27]. Currently, there is no effective therapy available. Comprehensive management should include non-pharmacological approaches, as recommended for other rheumatic conditions like lupus and scleroderma [28].

The link between PM and IBM may arise from diagnostic confusion, with some PM patients potentially having IBM. OM was mainly in cluster 3, consisting predominantly of females with a high incidence of MHDs, reflecting known epidemiology in rheumatology [25, 29]. Interestingly, despite the differing physical comorbidities, clusters 2 (characterized by a high comorbidity burden) and 3 (marked by a high burden of MHDs) showed similarly poor outcomes in PROMIS assessments. This emphasizes the significant impact of MHDs on overall physical health, mental well-being and fatigue. However, functional status, as measured by the SF-10a, appeared to be less impaired in cluster 3, despite the high burden of MHDs.

The strength of our study is its use of the extensive COVAD survey, the most ethnically and geographically diverse dataset of IIM patients to date. However, we acknowledge limitations such as convenience sampling, recall bias and reliance on unverified self-reported data.

To mitigate this, our survey underwent extensive pilot testing and validation across multiple languages and regions to ensure clarity and comprehensibility. Additionally, we included questions verified by rheumatologists to improve the accuracy of the reported diagnoses. There is the possibility that some patients with IBM may have been misclassified as having PM. Most patients with IIMs came from countries with very high HDI, and some underdiagnosed comorbidities from developing countries might have introduced some bias [30, 31]. However, we attempted to correct for this discrepancy by adjusting all analyses for HDI. Furthermore, the scope of the survey was limited to specific comorbidities, potentially leading to an underestimate of total comorbidity burden. It should be noted that in the COVAD database, ILD was recorded as a comorbidity, although it is recognized as a possible manifestation of the disease itself. To avoid bias, since it is assessed within the database context as a comorbidity, we have chosen to retain this involvement as a comorbidity in our analysis. However, we conducted a sub-analysis excluding ILD as a comorbidity for all AIRDs, which confirmed the results of a higher risk of comorbidities, BM and CM for IIMs. In this context, only for ASS do we observe a reversal of the risk of having at least one comorbidity. To bolster the credibility of our findings, future research should aim to include verification through hospital record linkage and a broader range of evaluated conditions to provide a more comprehensive understanding of the comorbidity burden in IIM patients.

In conclusion, our study underscores a substantial burden of comorbidities, including MHDs, among individuals with IIMs. Whether assessed by the presence of multiple concurrent conditions, the involvement of various organs/systems or mental health, these comorbidities significantly affect various facets of patients' lives. Therefore, the clinical management of IIM patients must adopt a comprehensive perspective that recognizes the influence of comorbidities and embraces a holistic approach to patient care.

## Supplementary Material

keae520_Supplementary_Data

## Data Availability

The datasets generated and/or analysed during the current study are not publicly available but are available from the corresponding author upon reasonable request.
