# Supplementary material for: The impact of multimorbidity on Quality of Life in inflammatory myopathies: A cluster analysis from the COVAD dataset
Source: Rheumatology (Oxford). 2024 Sep 25;64(4):2133–42. doi: 10.1093/rheumatology/keae520 (PMC11962953; doi:10.1093/rheumatology/keae520)
Supplement: keae520_Supplementary_Data [file keae520_supplementary_data.zip › keae520_Supplementary_Data/rhe-24-0573-File008.docx]

**Supplementary table 1: Recorded Comorbidities in COVAD-2 Survey**

| **Comorbidities’ category** | **Specific Comorbidity Recorded in COVAD-2 Survey** |
| --- | --- |
| Lung-Related Comorbidities | Asthma, COPD, ILD |
| Kidney-Related Comorbidity | CKD |
| Liver-Related Comorbidity | CLD |
| Heart-Related Comorbidities | IHD, Hypertension, Dyslipidemia |
| Central Nervous System-Related | Stroke, Epilepsy |
| Multiorgan-Related Comorbidities | Diabetes, HIV-AIDs |
| Mental Health Disorders | Anxiety, Bipolar Disorder, Depression, Eating Disorders, Insomnia, Schizophrenia, Substance Abuse |

Abbreviations: HIV-AIDs: Human Immunodeficiency Virus-Acquired Immunodeficiency Syndrome, IHD: Ischemic Heart Disease, CLD: Chronic liver disease, CKD: Chronic kidney disease, COPD: Chronic Obstructive Pulmonary Disease

**Supplementary table 2. PROMIS scores**

|  | **Questions** | **Options and scoring** |
| --- | --- | --- |
| **Physical function - Short Form 10a (SF10)**  PROMIS® Item Bank v1.0  (range: 0-50) | Does your health now limit you in doing vigorous activities, such as running, lifting heavy objects, participating in strenuous sports?  Does your health now limit you in doing moderate activities, such as moving a table, pushing a vacuum cleaner, bowling, or playing golf?  Does your health now limit you in lifting or carrying groceries?  Does your health now limit you in climbing several flights of stairs?  Does your health now limit you in climbing one flight of stairs?  Does your health now limit you in bending, kneeling, or stooping?  Does your health now limit you in walking more than a mile?  Does your health now limit you in walking several blocks?  Does your health now limit you in walking one block?  Does your health now limit you in bathing or dressing yourself? | Without any difficulty (5)  With a little difficulty (4)  With some difficulty (3)  With much difficulty (2)  Unable to do (1) |
| **Global Mental Health (PGM)**  PROMIS Scale v1.2  (range: 0-20) | In general, would you say your quality of life is:  In general, how would you rate your mental health, including your mood and your ability to think?  In general, how would you rate your satisfaction with your social activities and relationships? | Excellent (5)  Very Good (4)  Good (3)  Fair (2)  Poor (1) |
|  | How often have you been bothered by emotional problems such as feeling anxious, depressed or irritable? | Never (5)  Rarely (4)  Sometimes (3)  Often (2)  Always (1) |
| **Global Physical Health (PGP)**  PROMIS Scale v1.2  (range: 0-20) | In general, how would you rate your physical health?  To what extent are you able to carry out your everyday physical activities such as walking, climbing stairs, carrying groceries, or moving a chair? | Excellent (5)  Very Good (4)  Good (3)  Fair (2)  Poor (1) |
|  | How would you rate your pain on average? | From none to worst pain imaginable (0-10) |
|  | How would you rate your fatigue on average? | None (5)  Mild (4)  Moderate (3)  Severe (2)  Very severe (1) |
| **Fatigue 4a (SF4a)**  PROMIS Item Bank v1.0  (range: 0-20) | During the past 7 days…  I feel fatigued  I have troubles starting things because I am tired | Not at all (1)  At little bit (2)  Somewhat (3)  Quite a bit (4)  Very much (5) |
|  | In the past 7 days…  How run down did you feel on average?  How fatigued were you on average? | Not at all (1)  At little bit (2)  Somewhat (3)  Quite a bit (4)  Very much (5) |

**Supplementary table 3: Distribution of comorbidities in IIMs, oAIRDs and HCs for continents**

| **Europe** | IIMs (n=218) | oAIRDs (n=1754) | HCs  (n=668) | IIMs vs oAIRDs (OR [95%CI])* | IIMs vs HCs (OR [95%CI])* |
| --- | --- | --- | --- | --- | --- |
| Comorbidities overall, n(%) | 150 (68.8) | 915 (52.2) | 155 (23.2) | 1.75 (1.28-2.42) | 3.78 (2.55-5.60) |
| Basic multimorbidity, n(%) | 65 (29.8) | 362 (20.6) | 40 (6) | 1.40 (1.01-1.95) | 2.98 (1.79-4.96) |
| Complex multimorbidity, n(%) | 25 (11.5) | 140 (8) | 12 (1.8) | 1.35 (0.85-2.15) | 3.47 (1.51-7.94) |
| Mental health disorders, n(%) | 63 (28.9) | 564 (32.2) | 115 (17.2) | 0.85 (0.62-1.18) | 1.71 (1.10-2.64) |
| **North America** | IIMs (n=1028) | oAIRDs (n=650) | HCs  (n=945) | IIMs vs oAIRDs (OR [95%CI])* | IIMs vs HCs (OR [95%CI])* |
| Comorbidities overall, n(%) | 725 (70.5) | 326 (50.2) | 273 (28.9) | 1.23 (0.93-1.58) | 3.10 (2.14-4.47) |
| Basic multimorbidity, n(%) | 393 (38.2) | 138 (21.2) | 65 (6.9) | 1.20 (0.91-1.58) | 2.74 (2.24-6.25) |
| Complex multimorbidity, n(%) | 155 (15.1) | 62 (9.5) | 19 (2) | 1.09 (0.75-1.57) | 5.53 (2.39-12.84 |
| Mental health disorders, n(%) | 374 (36.4) | 255 (39.2) | 247 (26.1) | 1.12 (0.87-1.45) | 1.99 (1.35-2.95) |
| **Asia** | IIMs (n=135) | oAIRDs (n=1320) | HCs  (n=1151) | IIMs vs oAIRDs (OR [95%CI])* | IIMs vs HCs (OR [95%CI])* |
| Comorbidities overall, n(%) | 55 (40.7) | 455 (34.5) | 229 (19.9) | 1.22 (0.82-1.82) | 2.02 (1.29-3.16) |
| Basic multimorbidity, n(%) | 20 (14.8) | 145 (11) | 57 (5) | 1.41 (0.80-2.46) | 2.42 (1.22-4.78) |
| Complex multimorbidity, n(%) | 11 (8.1) | 47 (3.6) | 9 (0.8) | 2.51 (1.16-5.44) | 14.36 (4.38-47.07) |
| Mental health disorders, n(%) | 38 (28.1) | 209 (15.8) | 102 (8.9) | 1.90 (1.23-2.96) | 4.13 (2.47-6.91) |
| **Australia-South America-Africa** | IIMs (n=177) | oAIRDs (n=867) | HCs  (n=888) | IIMs vs oAIRDs (OR [95%CI])* | IIMs vs HCs (OR [95%CI])* |
| Comorbidities overall, n(%) | 120 (67.8) | 327 (37.7) | 257 (28.9) | 1.68 (1.08-2.62) | 2.46 (1.49-4.07) |
| Basic multimorbidity, n(%) | 67 (37.9) | 100 (11.5) | 64 (7.2) | 1.72 (1.03-2.89) | 2.58 (1.34-4.93) |
| Complex multimorbidity, n(%) | 27 (15.3) | 48 (5.5) | 10 (1.1) | 1.68 (0.84-3.34) | 3.77 (1.23-11.57) |
| Mental health disorders, n(%) | 55 (31.1) | 261 (30.1) | 168 (18.9) | 0.92 (0.59-1.45) | 1.68 (0.99-2.83) |

Abbreviations: CI: confidence interval; HCs: healthy controls; IIMs: idiopathic inflammatory myopathies; oAIRDs: other autoimmune rheumatic diseases; OR: odds ratio.

*Logistic analysis adjusted for Age, gender, ethnicity, HDI value

**Supplementary table 4: Distribution of comorbidities in IIMs, oAIRDs and HCs excluding interstitial lung disease.**

|  | IIMs (n=1558) | oAIRDs (n=4591) | HCs  (n=3652) | IIMs vs oAIRDs (OR [95%CI])* | IIMs vs HCs (OR [95%CI])* |
| --- | --- | --- | --- | --- | --- |
| Comorbidities overall, n(%) | 924 (59.3) | 1908 (41.6) | 897 (24.6) | 1.22 (1.06-1.39) | 2.00 (1.67-2.41) |
| Basic multimorbidity, n(%) | 460 (29.5) | 685 (14.9) | 223 (6.1) | 1.40 (1.20-1.64) | 2.70 (2.07-3.52) |
| Complex multimorbidity, n(%) | 193 (12.4) | 284 (6.2) | 52 (1.4) | 1.54 (1.24-1.92) | 4.88 (3.10-7.68) |

Abbreviations: CI: confidence interval; HCs: healthy controls; IIMs: idiopathic inflammatory myopathies; oAIRDs: other autoimmune rheumatic diseases; OR: odds ratio.

*Logistic analysis adjusted for Age, gender, ethnicity, HDI value

**Supplementary table 5. Distribution of comorbidities in different subsets of IIMs.**

|  | DM (n. 470) | PM (n. 217) | IBM (n. 375) | ASS (n. 103) | IMNM (n. 96) | OM (n. 239) |
| --- | --- | --- | --- | --- | --- | --- |
| **List of comorbidities, n. (%) and OR (95%CI)**  Asthma  CKD  CLD  COPD  ILD  IHD  Diabetes mellitus  Epilepsy  Dyslipidaemia  HIV-AIDS  Hypertension  Stroke  Tuberculosis  Organ transplant  Others  comorbidities | 65 (13.8)  **0.72 (0.52-0.99)**  10 (2.1)  ns  7 (1.5)  ns  12 (2.6)  **0.48 (0.24-0.95)**  95 (20.2)  ns  15 (3.2)  **0.44 (0.25-0.77)**  43 (9.1)  **0.66 (0.45-0.96)**  2 (0.4)  ns  92 (19.6)  **0.73 (0.55-0.97)**  0 (0)  ns  130 (27.7)  ns  6 (1.3)  ns  0 (0)  ns  1 (0.2)  ns    36 (7.7)  ns | 38 (17.5)  ns  10 (4.6)  ns  3 (1.4)  ns  13 (6)  ns  52 (24)  **1.71 (0.19-2.43)**  18 (8.3)  ns  42 (19.4)  **1.74 (1.18-2.56)**  1 (0.5)  ns  67 (30.9)  **1.38 (1.0-1.92)**  0 (0)  ns  87 (40.1)  **1.39 (1.02-1.92)**  2 (0.9)  ns  0 (0)  ns  0 (0)  ns  32 (14.7)  **2.25 (1.45-3.49)** | 44 (11.7)  ns  15 (4)  ns  6 (1.6)  ns  13 (3.5)  ns  3 (0.8)  **0.02 (0.01-0.72)**  58 (15.5)  ns  61 (16.3)  ns  1 (0.3)  ns  119 (31.7)  ns  5 (1.3)  **11.9 (1.2-119.4)**  156 (41.6)  ns  10 (2.7)  ns  2 (0.5)  ns  0 (0)  ns  26 (6.9)  **0.49 (0.29-0.82)** | 16 (15.5)  ns  1 (1)  ns  1 (1)  ns  2 (1.9)  ns  71 (68.9)  **14.10 (8.7-22.8)**  2 (1.9)  ns  10 (9.7)  ns  0 (0)  ns  16 (15.5)  ns  0 (0)  ns  23 (22.3)  ns  4 (3.9)  **3.83 (1.21-12.2)**  0 (0)  ns  1 (1)  ns  2 (1.9)  **0.24 (0.06-0.98)** | 19 (19.8)  ns  4 (4.2)  ns  1 (1)  ns  3 (3.1)  ns  2 (2.1)  **0.09 (0.02-0.36)**  10 (10.4)  ns  27 (28.1)  **3.07 (1.87-5.05)**  1 (1)  ns  38 (39.6)  **2.34 (1.49-3.65)**  0 (0)  ns  34 (35.4)  ns  3 (3.1)  ns  0 (0)  ns  0 (0)  ns  8 (8.3)  ns | 36 (15.1)  ns  12 (5)  ns  10 (4.2)  **3.06 (1.14-8.25)**  12 (5)  ns  31 (13)  **0.59 (0.37-0.93)**  15 (6.3)  ns  20 (8.4)  ns  3 (1.3)  ns  42 (17.6)  ns  1 (0.4)  ns  57 (23.8)  ns  1 (0.4)  ns  3 (1.3)  ns  0 (0)  ns  16 (6.7)  ns |
| **List of Mental health disorders, n. (%) and OR (95%CI)**  Anxiety  Bipolar disorder  Depression  Eating disorder  Insomnia  Schizophrenia  Substance use disorders | 111 (23.6)  ns  2 (0.4)  ns  94 (20)  ns  6 (1.3)  ns  43 (9.1)  ns  0 (0)  ns  0 (0)  ns | 47 (21.7)  ns  4 (1.8)  ns  46 (21.2)  ns  3 (1.4)  ns  21 (9.7)  ns  0 (0)  ns  0 (0)  ns | 69 (18.4)  **1.43 (1.0-2.05)**  0 (0)  ns  59 (15.7)  ns  1 (0.3)  ns  25 (6.7)  ns  1 (0.3)  ns  3 (0.8)  ns | 25 (24.3)  ns  0 (0)  ns  18 (17.5)  ns  1 (1)  ns  3 (2.9)  **0.27 (0.08-0.88)**  0 (0)  ns  0 (0)  ns | 20 (20.8)  ns  2 (2.1)  ns  22 (22.9)  ns  2 (2.1)  ns  6 (6.3)  ns  0 (0)  ns  0 (0)  ns | 47 (21.7)  ns  5 (2.1)  ns  56 (23.4)  ns  6 (2.5)  ns  36 (15.1)  ns  1 (0.4)  ns  3 (1.3)  ns |

Abbreviations: ASS: antisynthetase syndrome; CI: confidence interval; CLD: chronic liver disease; CKD: chronic kidney disease; COPD: chronic obstructive pulmonary disease; DM: dermatomyositis; HCs: healthy controls; HIV-AIDS: Human Immunodeficiency Virus-Acquired Immune Deficiency Syndrome ; IBM: inclusion body myositis; IMNM: immune mediated necrotizing myopathy; IHD: ischemic heart disease; IIMs: idiopathic inflammatory myopathies; ILD: interstitial lung disease (ILD); oAIRDs: other autoimmune rheumatic diseases; OM: overlap myositis; OR: odds ratio; PM: polymyositis

Logistic analysis (bold letters) adjusted for Age, gender, ethnicity, HDI value is reported as OR (95% CI)

**Supplementary table 6: Distribution of comorbidities, basic multimorbidity and complex multimorbidity in different subsets of IIMs excluding interstitial lung disease.**

|  | DM (n. 470) | PM (n. 217) | IBM (n. 375) | ASS (n. 103) | IMNM (n. 96) | OM (n. 239) |
| --- | --- | --- | --- | --- | --- | --- |
| Comorbidities overall, n(%)  **OR (95%CI)** | 237 (50.4)  **0.65 (0.51-0.83)** | 155 (71.4)  **1.87 (1.33-2.61)** | 268 (71.5)  ns | 45 (43.7)  **0.64 (0.42-0.99)** | 66 (68.6)  **1.75 (1.09-2.82)** | 121 (50.6)  ns |
| Basic multimorbidity, n(%)  **OR (95%CI)** | 100 (21.3)  **0.60 (0.45-0.78)** | 79 (36.4)  **1.40 (1.02-1.92)** | 151 (40.3)  ns | 18 (17.5)  ns | 42 (43.8)  **2.22 (1.43-3.45)** | 55 (23)  ns |
| Complex multimorbidity, n(%)  **OR (95%CI)** | 40 (8.5)  **0.51 (0.35-0.75)** | 32 (14.7)  ns | 47 (12.5)  ns | 8 (7.8)  ns | 23 (24)  **2.38 (1.43-3.96)** | 33 (13.8)  ns |

Abbreviations: CI: confidence interval; IIMs: idiopathic inflammatory myopathies; DM: dermatomyositis; IBM: inclusion body myositis; IMNM: immune mediated necrotizing myopathy; OM: overlap myositis; OR: odds ratio; PM: polymyositis

*Logistic analysis adjusted for Age, gender, ethnicity, HDI value
