# Supplementary figures and images for: The impact of multimorbidity on Quality of Life in inflammatory myopathies: A cluster analysis from the COVAD dataset
Source: Rheumatology (Oxford). 2024 Sep 25;64(4):2133–42. doi: 10.1093/rheumatology/keae520 (PMC11962953; doi:10.1093/rheumatology/keae520)

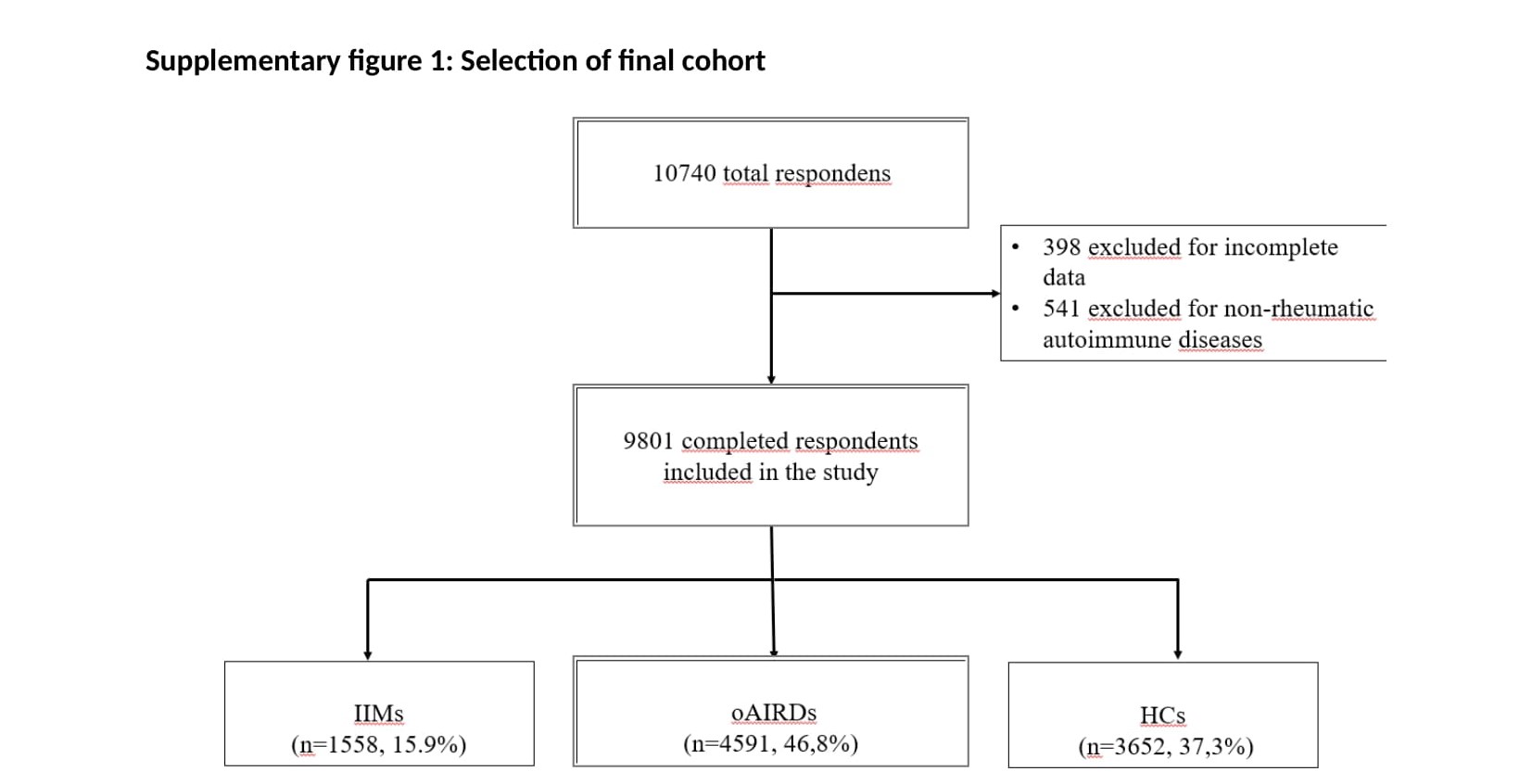

Supplement: keae520_Supplementary_Data [file keae520_supplementary_data.zip › keae520_Supplementary_Data/rhe-24-0573-File003.jpg]

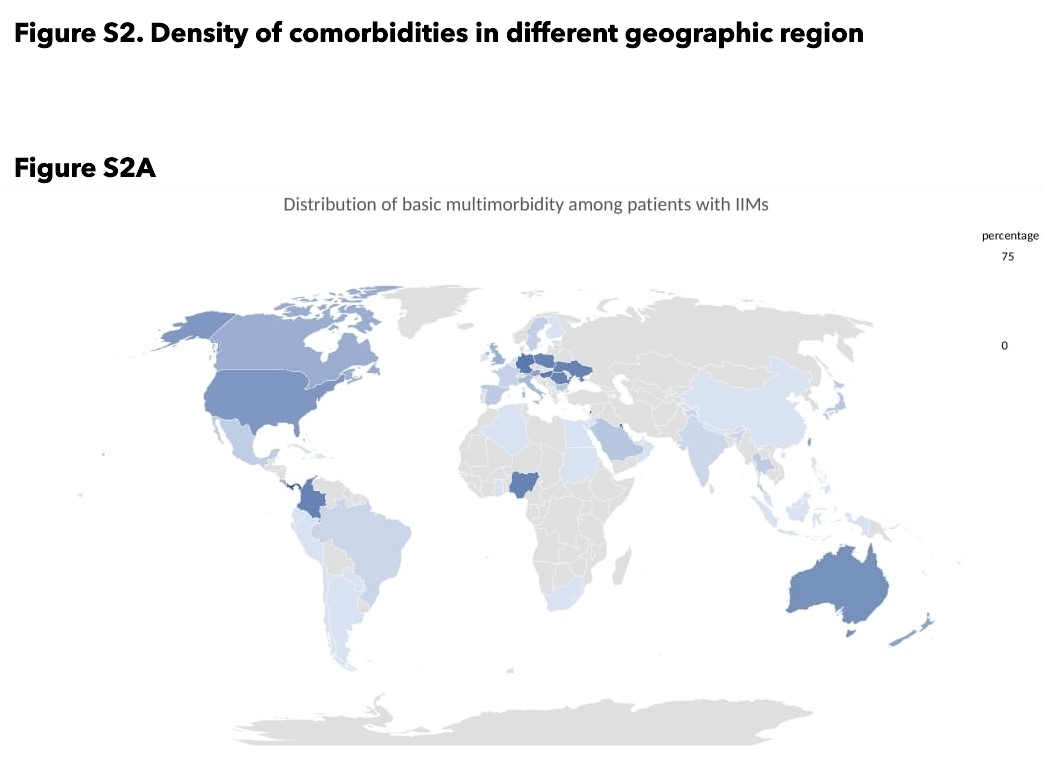

Supplement: keae520_Supplementary_Data [file keae520_supplementary_data.zip › keae520_Supplementary_Data/rhe-24-0573-File004.png]

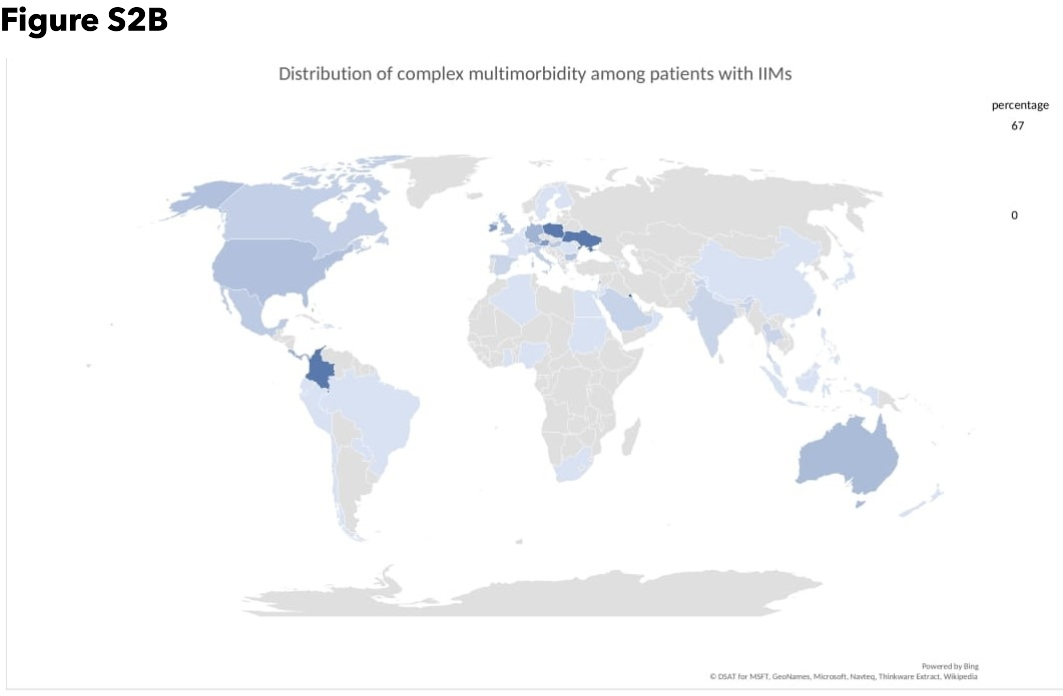

Supplement: keae520_Supplementary_Data [file keae520_supplementary_data.zip › keae520_Supplementary_Data/rhe-24-0573-File005.png]

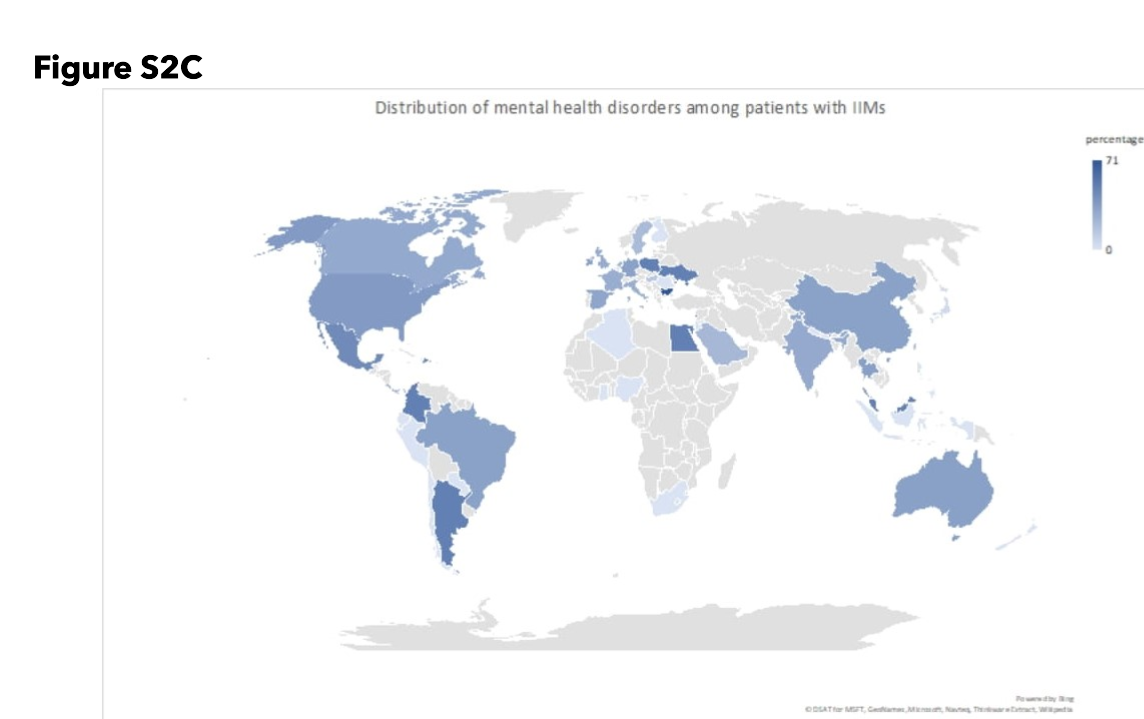

Supplement: keae520_Supplementary_Data [file keae520_supplementary_data.zip › keae520_Supplementary_Data/rhe-24-0573-File006.png]

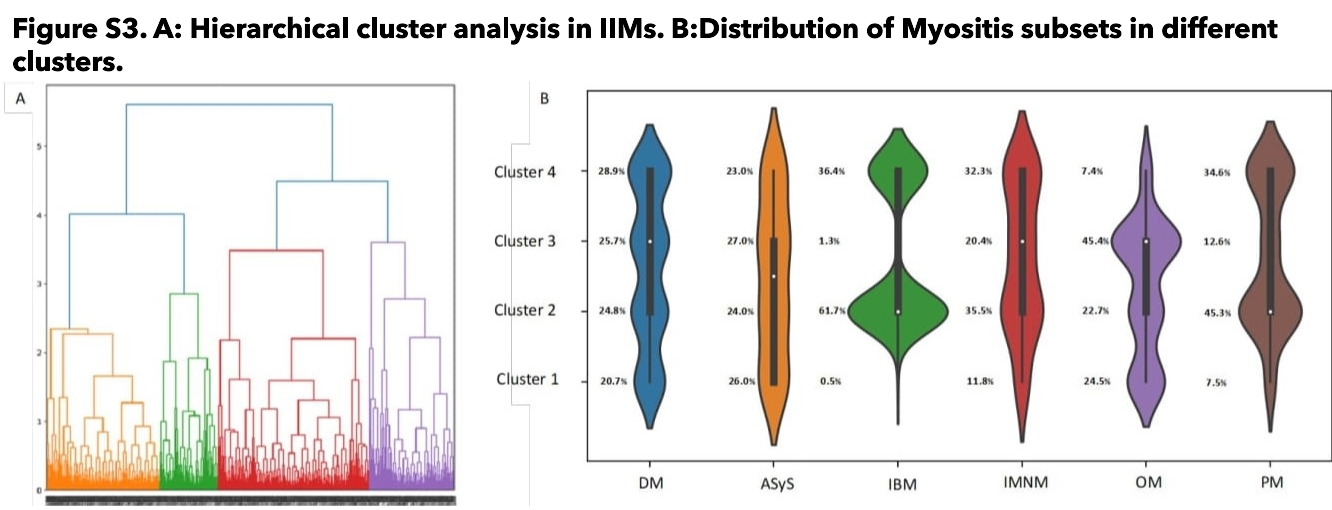

Supplement: keae520_Supplementary_Data [file keae520_supplementary_data.zip › keae520_Supplementary_Data/rhe-24-0573-File007.png]
